# GaMF1.39’s antibiotic efficacy and its enhanced antitubercular activity in combination with clofazimine, Telacebec, ND-011992, or TBAJ-876

**DOI:** 10.1128/spectrum.02282-23

**Published:** 2023-11-20

**Authors:** Priya Ragunathan, Pearly Shuyi Ng, Samsher Singh, Wee Han Poh, Dennis Litty, Nitin Pal Kalia, Simon Larsson, Amaravadhi Harikishore, Scott A. Rice, Philip W. Ingham, Volker Müller, Garrett Moraski, Marvin J. Miller, Thomas Dick, Kevin Pethe, Gerhard Grüber

**Affiliations:** 1 School of Biological Sciences, Nanyang Technological University, Singapore, Singapore; 2 Experimental Drug Development Centre, Agency for Science, Technology and Research, Singapore, Singapore; 3 Lee Kong Chian School of Medicine, Nanyang Technological University, Experimental Medicine Building, Singapore, Singapore; 4 Singapore Centre for Environmental Life Sciences Engineering, Nanyang Technological University, Singapore, Singapore; 5 Molecular Microbiology and Bioenergetics, Institute for Molecular Biosciences, Johann Wolfgang Goethe University Frankfurt/Main, Frankfurt, Germany; 6 Department of Biological Sciences (Pharmacology & Toxicology), National Institute of Pharmaceutical Education and Research, Hyderabad, Telangana, India; 7 School of Chemistry, Chemical Engineering and Biotechnology, Nanyang Technological University, Singapore, Singapore; 8 Department of Chemistry and Biochemistry, Montana State University, Bozeman, Montana, USA; 9 Department of Chemistry and Biochemistry, University of Notre Dame, Notre Dame, Indiana, USA; 10 Center for Discovery and Innovation, Hackensack Meridian Health, Nutley, New Jersey, USA; 11 Department of Medical Sciences, Hackensack Meridian School of Medicine, Nutley, New Jersey, USA; 12 Department of Microbiology and Immunology, Georgetown University, Washington, DC, USA; 13 National Centre for Infectious Diseases (NCID), Jalan Tan Tock Seng, Singapore, Singapore; Johns Hopkins University School of Medicine, Baltimore, Maryland, USA

**Keywords:** bioenergetics, *Mycobacterium tuberculosis*, tuberculosis, F-ATP synthase, oxidative phosphorylation, anti-TB compound

## Abstract

The *Mycobacterium tuberculosis* (*Mtb*) F-ATP synthase generates most of the biological energy currency ATP. Previously, we identified the mycobacterium-specific loop of the F-ATP synthase subunit γ as a new anti-tuberculosis target and discovered the novel diaminopyrimidine GaMF1, whose potency was improved by structure-activity relationship studies leading to the analog GaMF1.39. Here, we report that GaMF1.39 depletes cellular ATP formation by targeting the mycobacterial F-ATP synthase without affecting proton coupling or oxygen consumption. The antimycobacterial compound is bactericidal and potent against *Mtb* in macrophages without inducing phenotypic changes in biofilm formation, planktonic bacteria, or being toxic to zebrafish larvae. Combining GaMF1.39 with the NADH dehydrogenase inhibitor clofazimine, the cyt-*bcc:aa_3_* inhibitor Telacebec, or the F-ATP synthase inhibitor TBAJ-876 showed enhanced whole ATP synthesis inhibition and anti-tuberculosis activity. These results suggest that GaMF1.39 may add value to a compound combination targeting oxidative phosphorylation for tuberculosis treatment.

**IMPORTANCE:**

New drugs are needed to combat multidrug-resistant tuberculosis. The electron transport chain (ETC) maintains the electrochemical potential across the cytoplasmic membrane and allows the production of ATP, the energy currency of any living cell. The mycobacterial engine F-ATP synthase catalyzes the formation of ATP and has come into focus as an attractive and rich drug target. Recent deep insights into these mycobacterial F_1_F_O_-ATP synthase elements opened the door for a renaissance of structure-based target identification and inhibitor design. In this study, we present the GaMF1.39 antimycobacterial compound, targeting the rotary subunit γ of the biological engine. The compound is bactericidal, inhibits infection *ex vivo*, and displays enhanced anti-tuberculosis activity in combination with ETC inhibitors, which promises new strategies to shorten tuberculosis chemotherapy.

## INTRODUCTION

**FIG 1:**
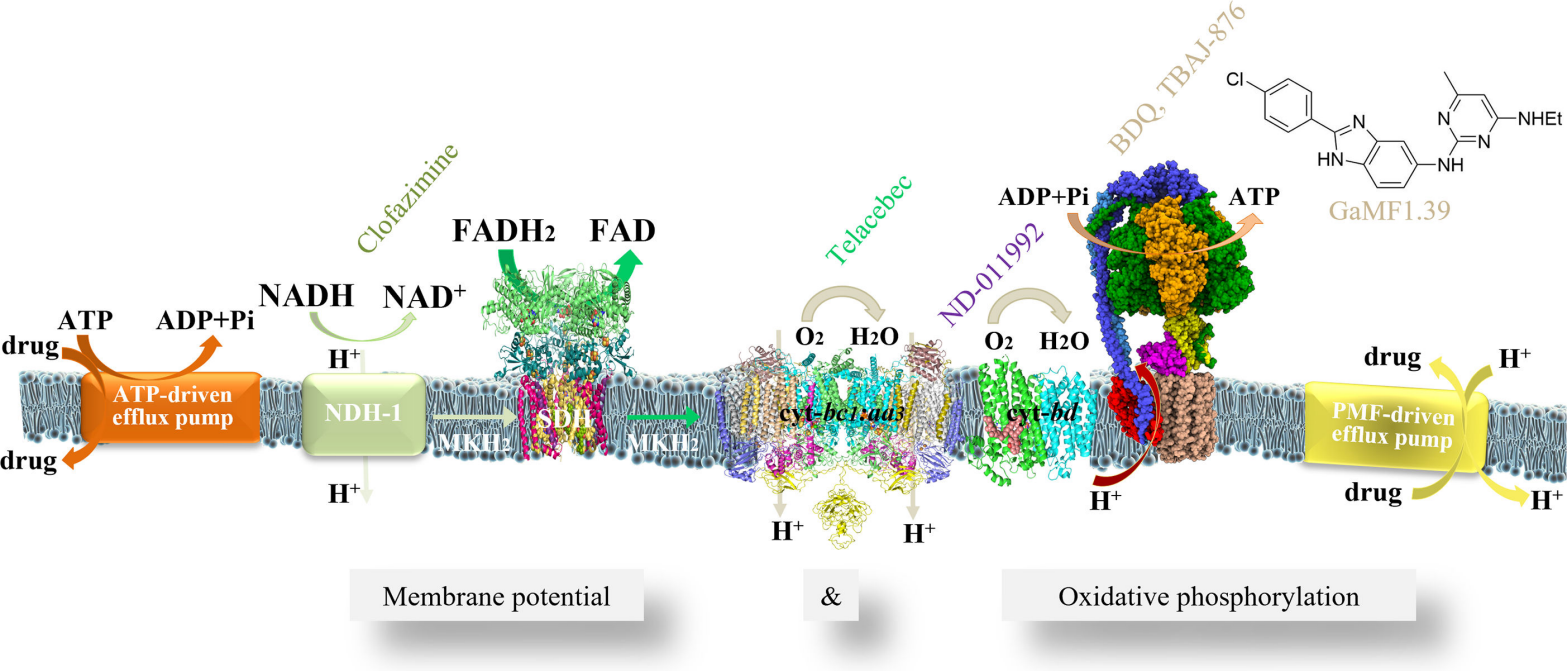
Enzyme composition and inhibitors of the mycobacterial ETC and F-ATP synthase. The cryo-electron microscopy structures of the *Mycobacterium smegmatis* (*Ms*) fumarate reductase (PDB ID: 6LUM), the cyt-*bc1:aa3* supercomplex (PDB ID: 7RH5), the *Mtb* cyt-*bd* (PDB ID: 7NKZ), and the *Ms* F-ATP synthase (PDB ID: 7NJK) are shown.

Tuberculosis (TB) is one of the major bacterial-caused infectious diseases and resulted in 1.5 million deaths in 2020, which, for the first time in over a decade, has increased according to the World Health Organization’s 2021 Global TB report (1). Multidrug-resistant and extensively drug-resistant bacterial strains of *Mycobacterium tuberculosis* (*Mtb*), which causes TB, are spreading worldwide, causing major global health issues. Drug tolerance of *Mtb* is proposed to be due to the ability of the pathogen to enter a metabolically quiescent state, in which it is phenotypically tolerant to drug challenge with conventional chemotherapeutics (2). In 2012, the anti-tuberculosis diarylquinoline bedaquiline (BDQ) was approved, which kills metabolically quiescent cells with delay by targeting the mycobacterial F_1_F_O_ ATP synthase and bringing oxidative phosphorylation (OXPHOS) and the electron transport chain (ETC) complexes (Fig. 1) into the focus of anti-TB drug development (3 – 10). However, the development of clinical resistance to BDQ has been reported (11), along with a very long terminal half-life leading to concerns regarding tissue accumulation and a study showing BDQ-inhibition of mitochondrial F-ATP synthase in human HEK293S cell mitoplasts (12). The anti-TB compound pipeline has lately been extended to provide a range of new scaffolds of mycobacterial F-ATP synthase inhibitors, including squaramides, dihydropyrazolo[1,5a]pyrazin-4-ones, diaminoquinazolines, thiazolidinediones, chloroquinolines, tetrahydroquinolines, diaminopyrimidines, new BDQ-analogs, including TBAJ-587 and -876, reviewed in 5, as well as new compound targets (9, 13 – 16).

The mycobacterial F_1_F_O_ ATP synthase, which is a latent ATPase and essential for growth (15, 17), consists of the subunits α_3_:β_3_:γ:ε:*b-δ:b′:a:c_9_* (18). Its H^+^-translocating F_O_ domain (subunits *a:c_9_*) uses the proton motive force (PMF), generated by the ETC complexes NADH dehydrogenase (in the case of NDH-1) and cytochrome *bcc:aa_3_* (cyt-*bcc:aa_3_*), to drive rotation of the central stalk subunits γ-ε. The latter causes sequential binding, entrapment, and phosphorylation of ADP to ATP within the nucleotide-binding and catalytic α_3_:β_3_-headpiece. The peripheral stalk subunits *b-δ:b*′ smooth the transmission of power between the rotary *c*-ring and the α_3_:β_3_:γ:ε domain (19).

Mycobacterial-specific modifications of the F-ATP synthase subunits α, δ, and γ, including a C-terminal elongation (14, 20, 21), an inserted domain (18), or a 12–14 amino acid extra loop (13), respectively, have been described as regulative (14, 20, 21) or essential features for catalysis (13, 14, 22). For example, the mycobacterial extra loop of subunit γ, absent in the human homolog and other prokaryotes, is important for ATP synthesis as well as ATP hydrolysis and ATP-driven proton pumping regulation (19) and has been identified as a new drug target (9, 13, 23). The most recent *Mycobacterium smegmatis* F-ATP synthase cryo-electron microscopy structure shows a conformation in which one of the polar γ-loop residues forms a salt bridge with an arginine residue of the peripheral stalk subunit *b′* during rotation (19). An *in silico* compound screening led to the discovery of the diaminopyrimidine GaMF1, targeting the mycobacterial γ-loop (9). Structure-activity relationship studies resulted in the analog 8 (now called GaMF1.39) that has an 18-fold improved activity compared to the parent compound (Fig. 1) (9).

Here, we present a detailed antimycobacterial activity study of the bactericidal GaMF1.39, evidence for its specific targeting of the mycobacterial F-ATP synthase, and anti-TB potency in macrophages, without altering biofilm formation or being toxic to zebrafish larvae. GaMF1.39 potentiates the anti-TB activity of the NADH dehydrogenase (NDH) inhibitor clofazimine (CFZ), the cyt-*bcc:aa_3_* inhibitor Telacebec (Q203) alone and in combination with the cyt-*bd* inhibitor ND-011992, as well as TBAJ-876, respectively.

## RESULTS AND DISCUSSION

### GaMF1.39 targets mycobacterial F-ATP synthase

**FIG 2:**
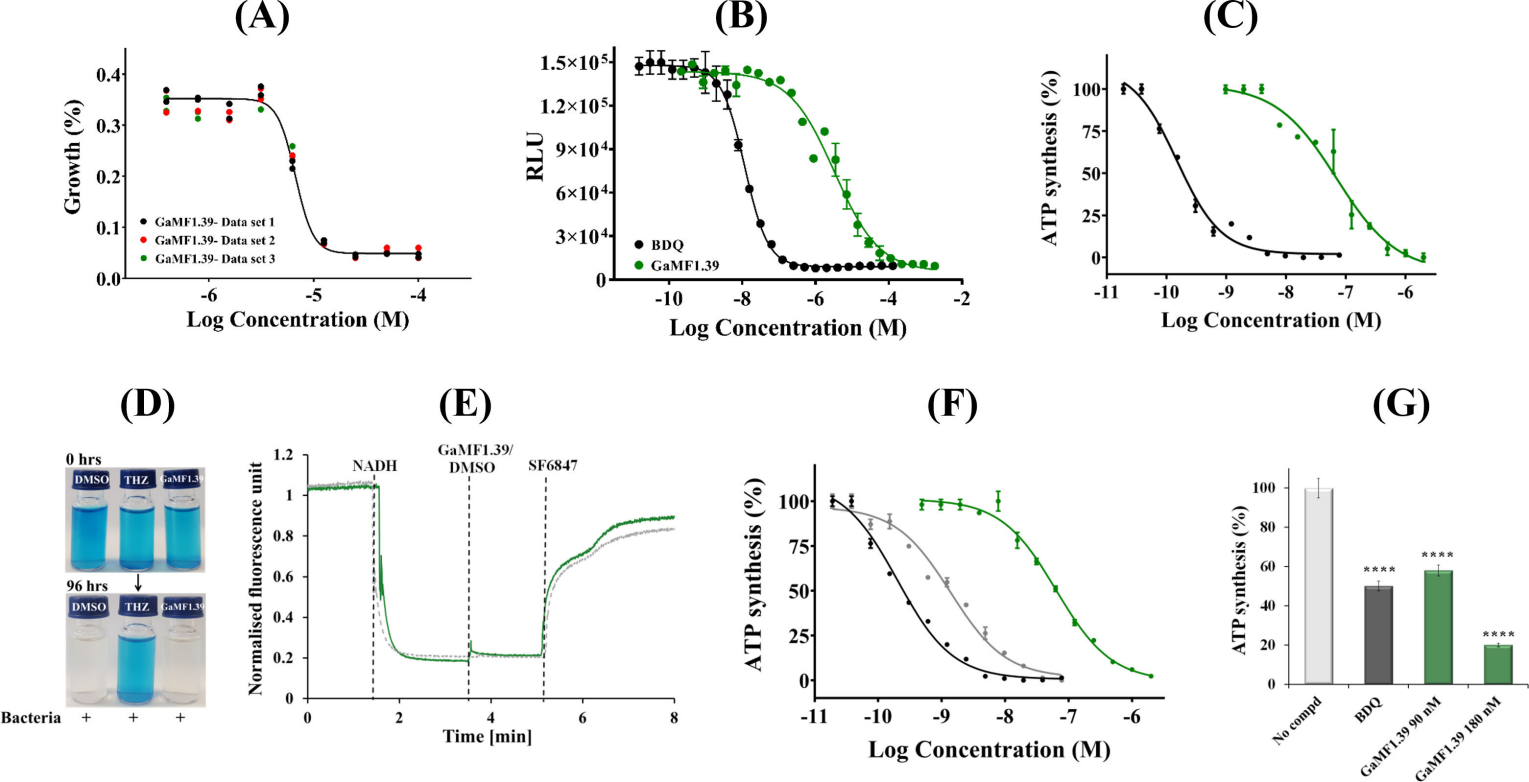
(**A**) Growth inhibition of *M. bovis* BCG cells by GaMF1.39. Growth curve of *M. bovis* BCG in the presence of an increasing amount of GaMF1.39 performed in 96-well flat-bottom cell culture plates. Suspension of *M. bovis* BCG in the logarithmic growth phase was added to obtain a starting OD_600_ of 0.05 in 200 µL. Plates were incubated for 5 days at 37°C. Three independent experiments were carried out, and the individual data points are shown in the graph. (**B**) Intracellular ATP synthesis of *M. bovis* BCG cells by GaMF1.39 (green) in comparison to BDQ (black). Shown is the effect of GaMF1.39 on the intrabacterial ATP content of whole-cell *M. bovis* BCG treated for 24 h. BDQ was used as a positive control. (**C**) Inhibition of ATP synthesis by GaMF1.39 in IMVs using the electron donor succinate (green). BDQ (black) was used as a control. ****, *P* < 0.0001; statistical analysis was carried out for the experiment using one sample *t*-test and a Wilcoxon test. (**D**) GaMF1.39 did not affect oxygen consumption in the slow-growing *M. bovis* BCG over a 96-h period. Thioridazine was used as a positive control, and 7H9 media with dimethyl sulfoxide (DMSO) as a solvent served as a blank or negative control. (**E**) GaMF1.39 does not function as an uncoupler. Effects of GaMF1.39 on the transmembrane pH gradient of mycobacterial IMVs. Then, 1 mM of GaMF1.39 (green) does not alter the quenching of the fluorescence of the pH-sensitive fluorophore 9-amino-6-chloro-2-methoxyacridine (ACMA). At the beginning of the experiments, 2 mM NADH, an electron donor, was added to the vesicle samples. The IMVs oxidized NADH and pumped protons to generate the transmembrane pH gradient, which was visualized as a quenching of fluorescence. Uncoupler SF6847 (1 µM) was added at the end of each experiment as a positive control to collapse the transmembrane pH gradient. The vertical dotted lines indicate the time points at which NADH, GaMF1.39, or SF6847 was added. The gray line shows the profile in the presence of DMSO as a control. (**F**) Inhibition of ATP synthesis by BDQ (gray) and GaMF1.39 (green) on *M. smegmatis* I66M *c*-subunit mutant IMVs with NADH as substrate. The effect of BDQ on wild-type IMVs (black) is shown as a control. (**G**) Effect of GaMF1.39 on the ATP synthesis of reconstituted *M. smegmatis* F-ATP synthase. The inhibitory effect of GaMF1.39 (90 and 180 nM) on *M. smegmatis* F-ATP synthase, which was reconstituted into proteoliposomes. BDQ at an IC_50_ of 4 nM was used in comparison. In the no-compound sample (DMSO), an end-concentration of 1.25% (vol/vol) was added to see whether DMSO has an effect on the ATP synthesis rate. The result represents two independent experiments (*n* = 2), performed at least in triplicate. ****, *P* < 0.0001; statistical analysis was carried out for the experiment using the ordinary one-way analysis of variance test.

As shown by the minimal inhibitory concentration (MIC_50_) of 6.8 µM and MIC_90_ of 12.2 µM (Fig. 2A), GaMF1.39 is a comparably efficacious inhibitor of *Mycobacterium bovis* bacillus Calmette–Guérin (BCG) and *Mtb* (3.0 µM) (9). The IC_50_ of 3.3 µM measured for intracellular ATP inhibition of *M. bovis* BCG (Fig. 2B) was consistent with the determined MIC_50_ and indicates that ATP formation, mainly synthesized by OXPHOS, is affected by the compound. NADH-driven ATP synthesis of inside-out membrane vesicles (IMVs) of *M. bovis* BCG and *M. smegmatis* was inhibited at an IC_50_ of 51.6 ± 1.35 and 90 ± 1.1 nM (9), respectively. In comparison, GaMF1.39 reduced the ATP synthesis of IMVs in the presence of succinate with a similar IC_50_ of 71 nM (Fig. 2C), indicating that the compound does not interfere with NADH dehydrogenases. Oxygen consumption within the respiratory chain of *M. bovis* BCG is unaffected at 7 µM of GaMF1.39 (Fig. 2D), and GaMF1.39 lacked the ability to induce uncoupled proton pumping (Fig. 2E), unlike the known uncoupler SF6847, underscoring that GaMF1.39 does not induce an increase in respiration as described for the anti-TB drug BDQ (24). In addition, we tested the potency of GaMF1.39 against *M. smegmatis* IMVs bearing an I66M substitution in subunit *c*, which is associated with resistance to BDQ (25). I66M mutant IMVs displayed a shift in sensitivity to BDQ with an IC_50_ and MIC_50_ value of 1 and 100 nM, respectively, in comparison to wild type (WT) (IC_50_ = 0.14 nM and MIC_50_ = 1 nM) (Fig. 2F, Fig. S1A), whereas the mutation did not affect the potency of GaMF1.39 (Fig. S1B). Similarly, when the resistant mutant strain εW16A was used, which leads to BDQ hypersusceptibility (26) (Fig. S1C), compound GaMF1.39’s potency did not change (Fig. S1D). These data suggest that GaMF1.39 shows no cross activity with BDQ on the enzyme complex and that GaMF1.39 is potent against BDQ resistant mutants.

Since GaMF1.39 lacked the ability to induce uncoupled proton pumping (Fig. 2E) and does not alter the membrane potential, we reconstituted the recombinant *Ms*F-ATP synthase into proteoliposomes to confirm that this molecular engine is indeed the target of the molecule. The advantage of this reconstitution design for compound screening lies not only in its efficiency but also in the fact that it does not need a second proton-motive force-generating enzyme, essential for target characterization. The reconstituted *Ms*F-ATP synthase had an ATP synthesis activity of 35.2 ± 2.3 nmol·min^−1^ (mg protein)^−1^ (Fig. 2G). When the control compound BDQ was used at its IC_50_ determined in IMVs (Fig. 2G), a 51% inhibition was observed, confirming that the engine is indeed BDQ’s target, suggesting that the assay is a useful tool for demonstrating compound target specificity. Similarly, when GaMF1.39 was tested against the reconstituted *Ms*F-ATP synthase at its IC_50_, a 42% inhibition (20.5 ± 2.6 nmol·min^−1^ (mg protein)^−1^) was calculated (Fig. 2G), and increasing the amount of GaMF1.39 resulted in a clear concentration-dependent inhibition of the enzyme, underscoring that the compound targets the enzyme.

### GaMF1.39 targets the mycobacterial γ-loop

**FIG 3:**
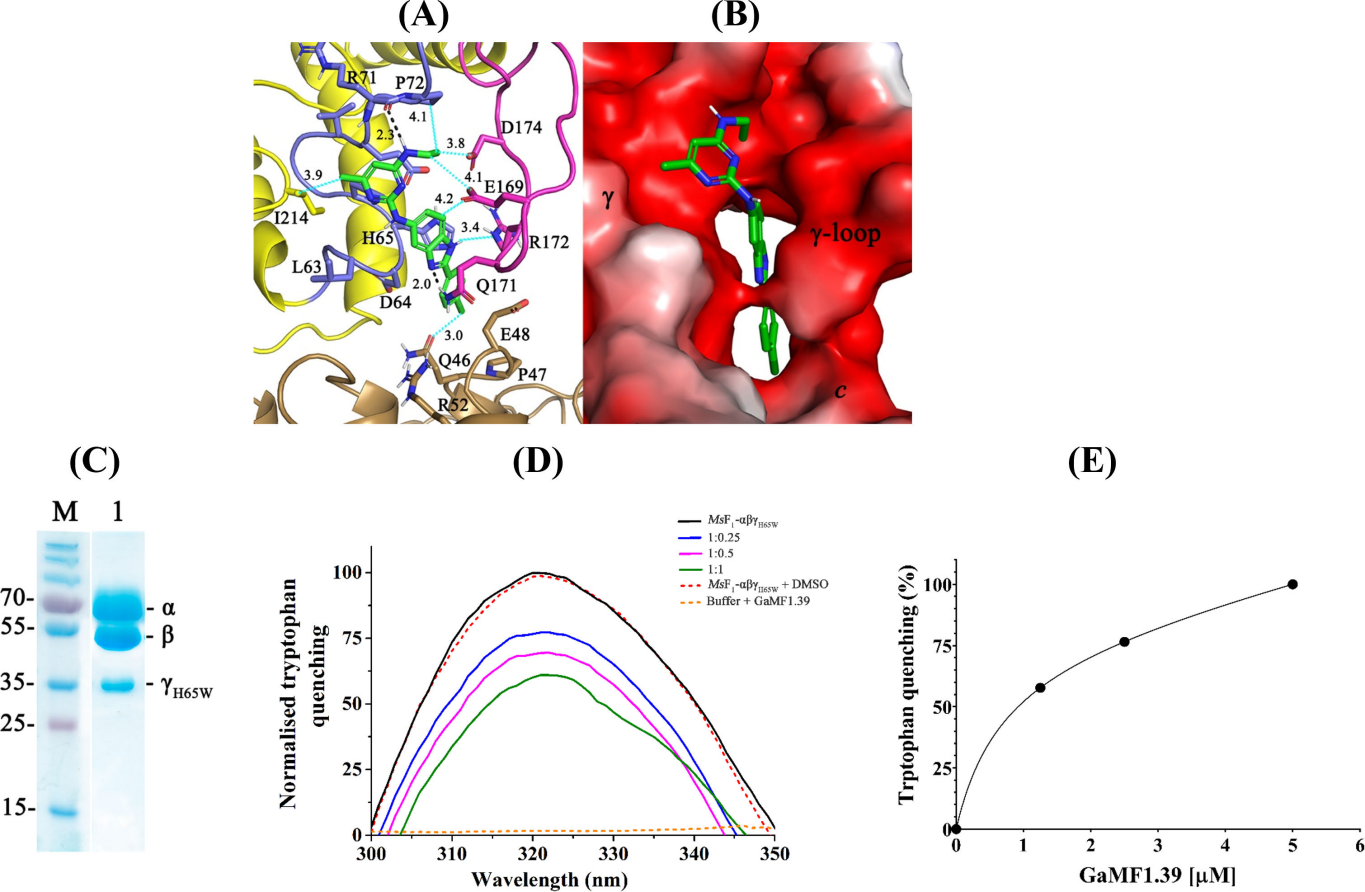
GaMF1-39 binds to the mycobacterial γ-loop. (**A**) GaMF1.39 was predicted to bind to *Mtb* subunit γ with a ChemPLP Fitness score of 49. The aminoethyl group of the pyrimidine ring has hydrogen bonding interactions (black lines, 2.3 Å) with R71 main chain amide atoms. While the 6-methyl group on pyrimidine (ring C) reveals an aliphatic interaction with amino acid I214 (3.9 Å, cyan dotted lines), the central ring, benzimidazole, with its nitrogen (N_2_) atom, mediates the H-bonded interaction with the γ-loop residue Q171 (2.0 Å, black lines). The benzimidazole ring was positioned to maintain van der Vaal’s contacts (cyan dotted lines) with H65 (4.6 Å), E169 (3.18 Å), and side chain amide atoms. The 4-chlorophenyl ring is in close proximity to the *c* subunit (light brown) residue Q46 (3.0 Å, cyan dotted lines). (**B**) Potential surface view of GaMF1.39 on *Mtb* subunit γ. (**C**) Purified *Ms*F_1_-αβγ_H65W_ mutant (lane 2) and a molecular weight standard (lane 1) on a 17% SDS gel. (**D**) Fluorescence emission spectra of *Ms*F_1_-αβγ_H65W_ (black line) with an emission maximum at 323 nm. An excitation wavelength of 295 nm was used. In comparison, the spectra in blue, magenta, and green reveal fluorescence quenching in the presence of GaMF1.39 with a molar ratio of *Ms*F_1_-αβγ_H65W_:GaMF1.39 of 1:0.25, 1:0.5, and 1:1, respectively, reflecting that the compound binds to *Ms*F_1_-αβγ_H65W_. The spectra of *Ms*F_1_-αβγ_H65W_ in the presence of the vehicle control DMSO (dashed red line) did not cause fluorescence quenching, confirming that GaMF1.39 targets mycobacterial subunit γ. In addition, the spectrum of GaMF1.39 (dashed orange line) alone had no effect on the tryptophan fluorescence quenching. (**E**) The binding constant of GaMF1.39 to *Ms*F_1_-αβγ_H65W_ was determined by tryptophan fluorescence quenching.

Recent NMR titration experiments and docking studies of the parent molecule GaMF1 demonstrated its binding to the *Mtb* γ-loop (9). To obtain further insights into the molecular interactions of the lead, GaMF1.39, with its three ring groups [4-chlorophenyl (ring A), 1H-benz[d]imidazole rings (ring B), and N^4^-ethyl-6-methylpyrimidine-2,4-diamine (ring C)], a molecular docking approach was performed (Fig. 3A–B). Overall, GaMF1.39 was predicted to dock in the vicinity of the *Mt*γ-loop residues with an improved ChemPLP fitness score of 49 when compared to its parent molecule’s fitness score of 46.6. On one hand, the amino linker of GaMF1.39, connecting the N^4^-ethyl group on pyrimidine (ring C), maintained the hydrogen bonding interactions with R71 main chain amide atoms (black dashed lines, 2.3 Å; Fig. 3A). While the N^4^-ethyl group on ring C was engaged in van der Waal’s contacts with γP72 (4.1 Å) and γD174 residues (3.8 Å) (Fig. 3A), likewise, the 6-methyl on ring C was anchored in aliphatic hydrophobic interactions with γI214 (3.9 Å, α2/β5 loop, cyan dotted lines). On the other hand, the benzimidazole nitrogen (N_2_) atom mediated the hydrogen-bonded interaction with γ-loop residue Q171 (black dashed lines, 2.0 Å). The benzimidazole ring was positioned to maintain van der Vaal’s contacts (cyan dotted lines) with H65 (4.8 Å, not shown for clarity), E169 (3.18 Å), and R172 side chain amide atoms (3.4 Å). Finally, the 4-chlorophenyl group (ring A) was also in close anchorage (3.26 Å, cyan dotted lines) to residue Q46 of the *c* subunit (light brown).

During 360° rotation, the γ-loop of the rotary γ subunit reaches one conformation, where it comes near the peripheral stalk subunit *b*′ (19). To test the possible binding of GaMF1.39 to the γ-loop while it reaches this confirmation, we performed an additional docking study on the γ-loop-*b*′ interface conformation, which revealed a lower ChemPLP fitness score of 36.2 for GaMF1.39 than the one described above (PLP fitness of 49), where the inhibitor is interacting mainly with the γ-loop residues and its surrounding subunit γ residues. The data underline that GaMF1.39 binds specifically to the mycobacterial γ-loop.

To underscore that GaMF1.39 targets the mycobacterial γ-loop and the docking data described, we designed a tryptophan mutation of residue γH65 (H65W) for binding affinity measurements. The γH65 residue lies in close proximity (4.6 Å) to the ligand binding site. Homology models by mutation of γH65 to tryptophan did not show any steric clashes with its neighboring residues or alter the secondary structure. Furthermore, this variation should not alter ligand binding as the closest carbon atoms of the indole ring of tryptophan residue are spaced at 2.33–2.35 Å from the chloro-benzene ring of GaMF1.39 (Fig. 3A). To avoid any possible effect of the tryptophan residues by subunits *a*, *b*, *b*′, δ, and ε, the recombinant *M. smegmatis* F_1_-αβγ_H65W_ (*Ms*F_1_-αβγ_H65W_) mutant was generated (Fig. 3C). The fluorescence spectrum of *Ms*F_1_-αβγ_H65W_ reveals a concentration-dependent quenching with a drastic drop at a 1:1 molar ratio of *Ms*F_1_-αβγ_H65W_:GaMF1.39 (Fig. 3D). As a control, DMSO (vehicle) was added to the protein, but no major change in fluorescence could be observed, demonstrating that GaMF1.39 targets the mycobacterial γ-loop. The fitted tryptophan-titration curve in Fig. 3E reflects a one-site binding mode, resulting in a dissociation constant (*K_D_*) of 0.7 ± 0.09 µM. Considering GaMF1.39’s 4-chlorophenyl group proximity to the *c* subunit residue Q46, which would be missing in our *Ms*F_1_-αβγ_H65W_:GaMF1.39 bound complex, the determined *K_D_* is in a similar range as the IC_50_, determined for the *M. smegmatis* IMVs (90 ± 1.1 nM) (9).

### GaMF1.39 is bactericidal

**FIG 4:**
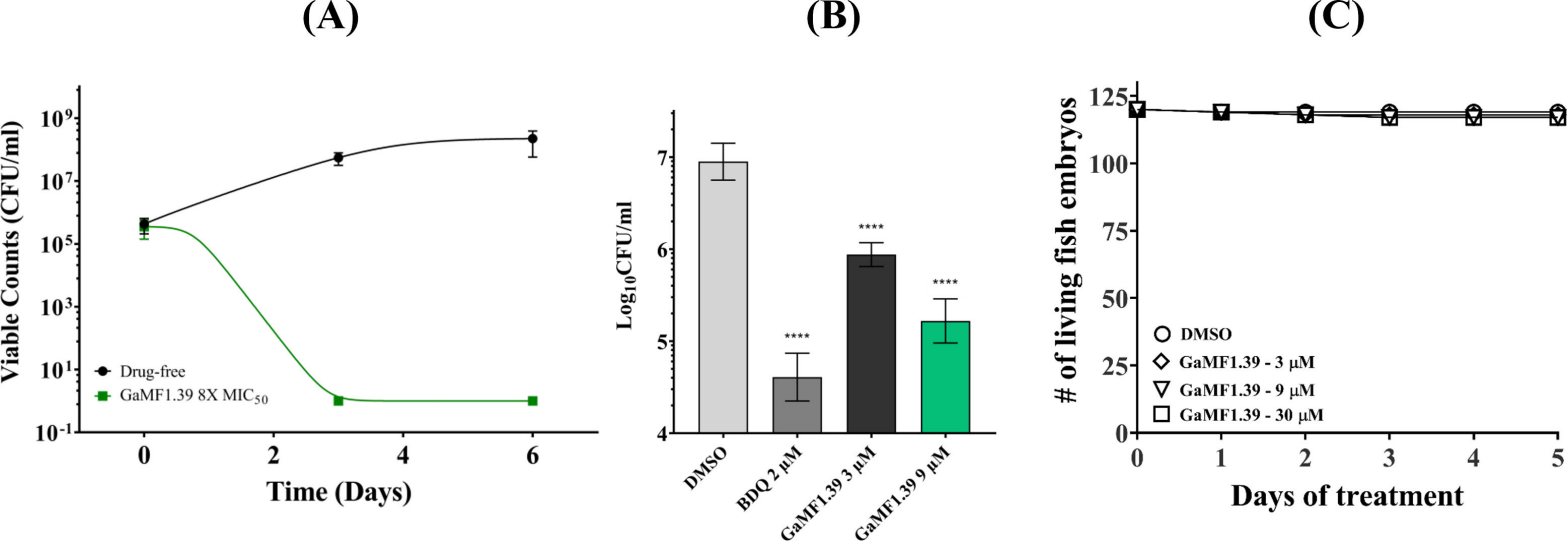
(**A**) Initial 6 days of untreated and GaMF1.39 kill kinetics against *M. bovis* BCG. The bacteria were grown in liquid culture Luria-Bertani broth supplemented with 0.05% (vol/vol) Tween 80 (LBT) in the presence of the indicated concentrations of GaMF1.39 for up to 6 days. Colony-forming unit was calculated by plating the culture on 7h10 agar plates. *, *P* < 0.05; statistical analysis was carried out for the experiment using the ordinary one-way analysis of variance (ANOVA) test. (**B**) Intracellular efficacy of GaMF1.39 on *M. tuberculosis* H37Rv-infected THP-1 cells. Macrophages were infected with *M. tuberculosis* H37Rv and treated with GaMF1.39 (3 µM, 9 µM), BDQ (2 µM), and DMSO. Experiments were repeated three times in triplicate and analyzed with the one-way ANOVA test; ****, *P* ≤ 0.0001. (**C**) Toxicity test of GaMF1.39 in zebrafish embryos; 48-hour post fertilization embryos were exposed to 3, 9, and 30 µM (10-fold MIC_50_ in *Mtb*) of GaMF1.39 dissolved in E3 medium for 5 days. The survival plot shows the number of live embryos or larvae upon exposure to GaMF1.39. DMSO was used as vehicle control. Two independent experiments were carried out, each with three technical replicates.

**FIG 5:**
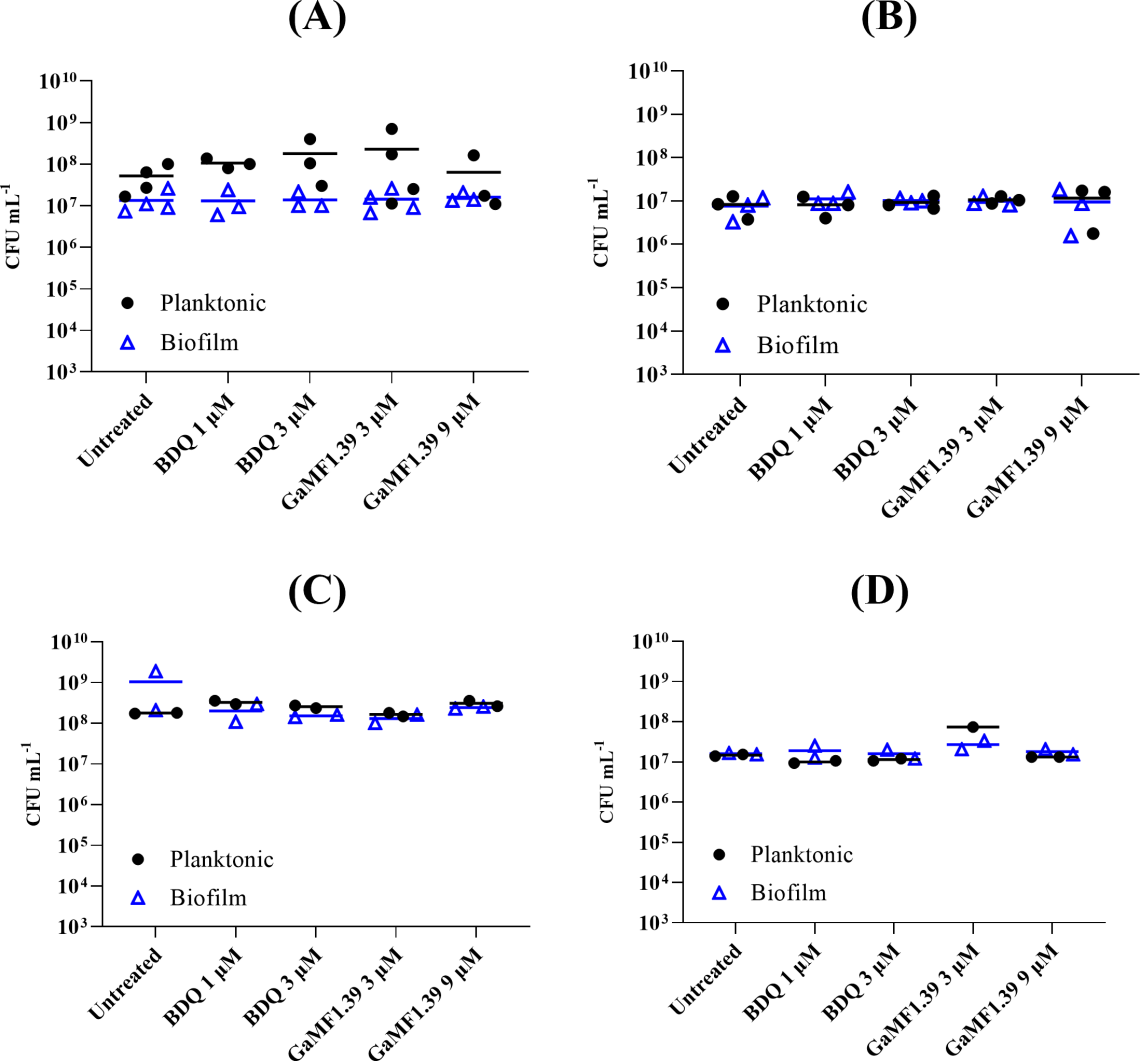
Activity of 1–3 µM of BDQ and 3–9 µM of GaMF1.39 against 3 h pre-formed biofilm of *P. aeruginosa* PAO1 WT (**A, C**) and *E. coli* UTI 189 (**B, D**). Treatment was carried out in 1x PBS media (**A, B**) or M9 glucose media (**C, D**). Both planktonic and biofilm cells were collected from the same sample well, with planktonic cells (black circles) referring to suspended cells present in treatment buffer prior to washing and collection of biofilm cells (open blue triangles). No significant differences were observed between GaMF1.39, the untreated controls, and the respective BDQ controls. At least two independent experiments were carried out, each with two technical replicates. Each data point represents the averaged data of an independent experiment, and the line represents the means of all the data.

The killing efficiency of GaMF1.39 against *M. bovis* BCG was tested at eightfold its MIC_50_, which is shown clearly by broth experiments and on 7H10 agar plates (Fig. S2A and B). At that concentration, GaMF1.39 (Fig. 4A) is more efficient than its parent molecule GaMF1 (9), and it did not show delayed bactericidal activity like BDQ as reported before (27). To explore the anti-TB potency of the compound in macrophages, a THP-1 infection model was used. As shown in Fig. 4B, GaMF1.39 was active against *M. tuberculosis* H37Rv at 3 and 9 µM and showed a decrease in viable bacterial count (CFU/mL) when compared with the initial inoculum (day 0). The infected macrophages did not show any visible optical alteration of the membrane, cell form, or size as tested microscopically. This is in line with experiments performed on zebrafish embryos, where no signs of toxicity-induced killing were recorded in the presence of 3, 9, or 30 µM (10× MIC_50_ against *Mtb*) of GaMF1.39 (Fig. 4C). Similarly, no killing of planktonic or biofilm cells was observed upon addition of 3–9 µM of GaMF1.39 to batch biofilms of *Pseudomonas aeruginosa* PAO1 WT or *Escherichia coli* UTI 189 under nutrient-limited conditions, simulated by treatment in phosphate buffered saline (PBS), and under growth conditions, simulated by the addition of fresh growth medium (Fig. 5A–D). These results suggest GaMF1.39’s target specificity and indicate that the compound is not a broad-spectrum antibacterial. A microsomal stability assay showed that GaMF1.39 is metabolically stable in mouse liver microsomes (*T*_1/2_ of 29.6 min, Cl_int_ of 46.8 mL/min/mg protein) while being slightly less lipophilic (clogP = 6.51) than BDQ (clogP = 7.25).

### Compound combinations inhibit the OXPHOS pathway

**FIG 6:**
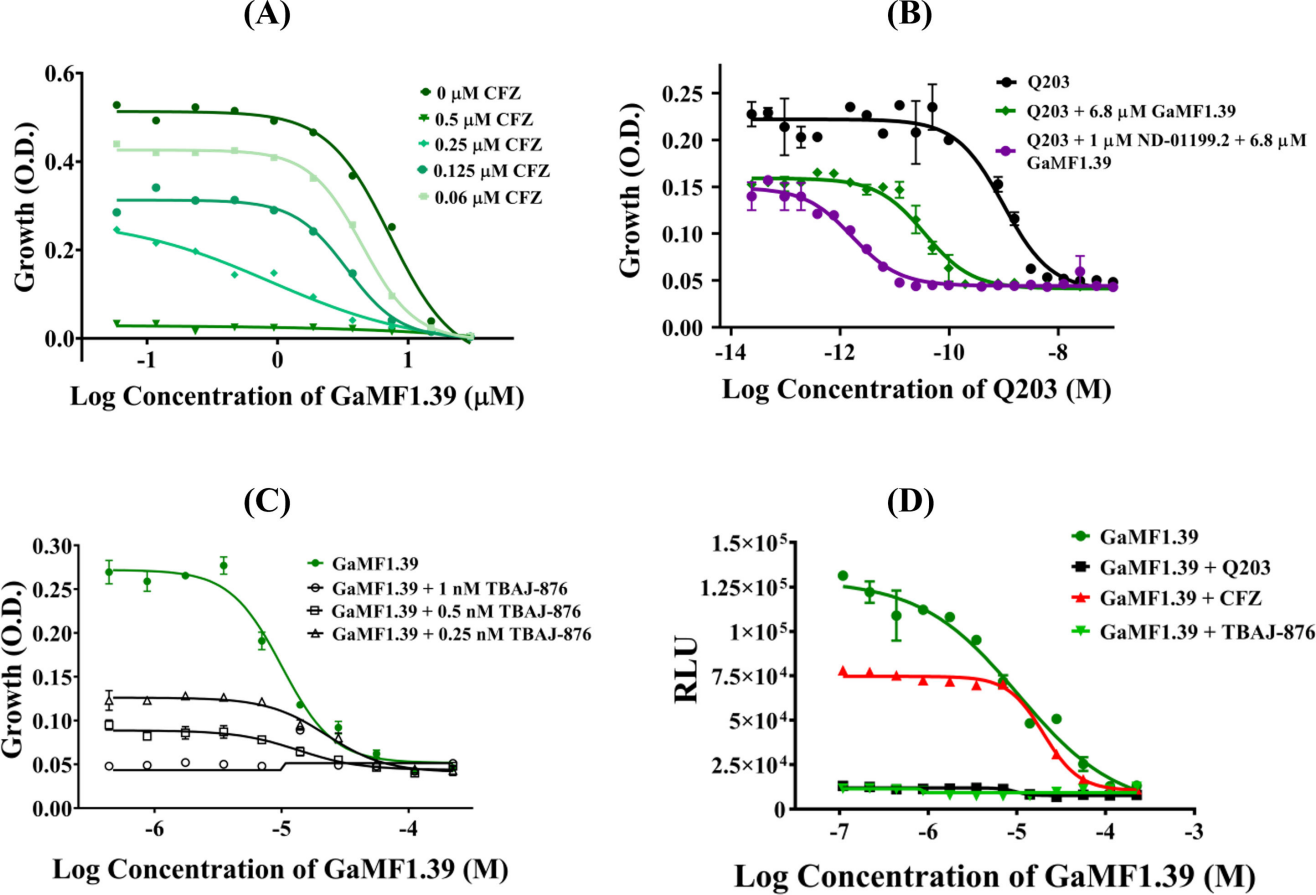
Enhanced potency of GaMF1.39 in combination with the antibiotics CFZ, Q203, ND-011992, or TBAJ-876. (**A**) GaMF1.39 (●) susceptibility of *M. bovis* BCG in combination with indicated concentrations of CFZ. (**B**) The inhibitory effect of GaMF1.39 (●) in combination with Q203 and ND-011992. (**C**) TBAJ-876 increases the potency of GaMF1.39 growth inhibition in *M. bovis* BCG. (**D**) Whole cell ATP depletion of the combinations GaMF1.39 + CFZ (0.5 µM), GaMF1.39 + Q203 (100 nM), or GaMF1.39 + TBAJ-876 (4 nM) in comparison to GaMF1.39 alone. ****, *P* < 0.0001; statistical analysis was carried out for all the above experiments using the ordinary one-way analysis of variance Bartlett’s test.

To reduce the viability of *Mtb*, the ETC has been demonstrated to be a druggable complex with modulators such as (i) CFZ, proposed to compete with the mycobacterial specific electron acceptor menaquinone for its reduction by the NDH-2 complex (28) and circumventing respiration by shuttling electrons from NADH directly to oxygen (29), (ii) the phase 2 drug candidate *bc1* cytochrome oxidase inhibitor Q203, which is bacteriostatic (8), or (iii) the anti-TB drug BDQ (4) and its 3,5-dialkoxypyridine analog TBAJ-876 (4), both inhibiting the final step of oxidative phosphorylation by inhibiting the mycobacterial F-ATP synthase. The treatment of TB requires drug combinations. As demonstrated for the *bc1*- and *bd* cytochrome oxidase inhibitors Q203 and ND-011992, with the latter being ineffective on its own, the combination improved intracellular ATP inhibition and was bactericidal (10). Here, we investigated the interaction of GaMF1.39 with CFZ, effecting the first complex of the ETC. As revealed in Fig. 6A, CFZ reduced the MIC_50_ of GaMF1.39 in a concentration-dependent manner. The checkerboard assay demonstrates an additive growth reduction by the combination of CFZ and GaMF1.39 (Fig. S3A), achieving a minimum fractional inhibitory concentration (FIC) index of 0.75 in *M. bovis* BCG (Table S1). In comparison, the combination of the *bc1* inhibitor Q203 and GaMF1.39 showed a significant growth reduction (Fig. 6B), which was further enhanced in the presence of the cyt-*bd* inhibitor ND-011992. The checkerboard assay revealed a slightly higher combinatory effect of GaMF1.39 + Q203 than of GaMF1.39 + CFZ, as shown in Fig. S3B and by the FIC index of 0.55 (Table S1). In addition, Q203 lowered the MIC_50_ of GaMF1.39 from 6.8 to 0.23 µM (Table S1). Since GaMF1.39 showed no cross resistance to BDQ (see above), we tested whether GaMF1.39 would antagonize or potentiate the activity of the clinical development candidate and BDQ analog TBAJ-876, which is less lipophilic (clogP = 5.80), has higher clearance, and displays lower cardiotoxic potential than BDQ (4). As shown in Fig. 6C, GaMF1.39’s potency was enhanced in the presence of different concentrations of TBAJ-876.

Growth inhibition of the regimen comprising GaMF1.39 + Q203 or GaMF1.39 + TBAJ-876 correlated well with the depletion of intracellular ATP (Fig. 6D), underscoring that the increased potency of the combinations was due to the reduction of OXPHOS. Interestingly, the decline in ATP formation of GaMF1.39 + CFZ was moderate. Since CFZ decreases the central carbon metabolism and PMF and generates reactive oxygen species (ROS) intracellularly (24), the data imply that the enhanced potency is partly due to ATP synthesis inhibition but may also be due to a reduced carbon metabolism and PMF as well as an increased formation of ROS.

Because of the lower FIC index (0.55) of the GaMF1.39 + Q203 than of the GaMF1.39 + CFZ combination, we tested whether the increase in growth reduction of the GaMF1.39 + Q203 regimen (Fig. 6B) would also increase the killing potency of GaMF1.39 (Fig. 3A). Fig. S4 reveals that GaMF1.39 and GaMF1.39 + Q203 inhibit the growth of *M. bovis* cells even after 10 days, showing that GaMF1.39 does not antagonize Q203 or increase the already existing killing potency of GaMF1.39.

### Conclusion

The enzymes of the ETC are responsible for recycling the reduced electron carriers NADH and FADH_2_ from central carbon metabolism, thereby facilitating redox balance and generating a PMF essential for maintaining transmembrane electrochemical gradients to regulate PMF-driven pumps and to drive ATP synthesis via OXPHOS (Fig. 1), processes that have been found to be required during persistence in *Mtb* (30 – 32). Both Q203 and BDQ are reported to induce reductions in ATP levels by increasing central carbon metabolism, NADH production, oxygen consumption rate, and, in the case of Q203, rerouting electron flux through cyt-*bd* oxidase, resulting in a bacteriostatic inhibition rather than killing activity (24). A detailed study of BDQ’s induced cell death has revealed the rerouting of glycolysis via the pentose phosphate pathway back to the energy payoff phase of glycolysis to conserve energy and increase ATP formation by substrate-level phosphorylation, in line with the delayed cidal effect of BDQ (15, 24). The bactericidal activity of BDQ is indistinguishable from that of the BDQ analog TBAJ-876 and is not affected by any uncoupling effect for both compounds (33). Targeting the mycobacterial F-ATP synthase GaMF1.39 depletes cellular ATP formation, required for replication, ana- and catabolic processes, cell wall formation, and ATP-dependent efflux pumps, which play a central role in resistance by expelling the respective prodrug or drug (Fig. 1). The compound does not affect oxygen consumption or H^+^-coupling and specifically inhibits the mycobacterial F-ATP synthase from generating ATP. Drug-drug potency interaction studies with the ETC- or OXPHOS inhibitors CFZ, Q203, ND-011992, and TBAJ-876 revealed no antagonistic effect, which is a prerequisite for combinatory approaches to treat *Mtb* infections. Furthermore, GaMF1.39’s efficacy increased in combination with CFZ, Q203, ND-011992, or TBAJ-876. GaMF1.39 is potent in mycobacterial cell death and against *Mtb*-infected macrophages without affecting zebrafish embryonic development or viability, opening the door for efficacy studies in *Mtb*-infected mouse models.

## MATERIALS AND METHODS

### Bacterial strains, culture medium, and chemicals

The *M. smegmatis* mc (2) 155 (ATCC 35734) and *M. bovis* BCG (ATCC 700084) strains were used in this study. The cultures were maintained in complete Middlebrook 7H9 medium (Sigma-Aldrich, St. Louis, MO, USA) supplemented with 0.5% (vol/vol) glycerol (Promega, Madison, WI, USA), 0.05% Tween-80 (Sigma-Aldrich), and 10% Middlebrook albumin-dextrose-catalase (ADC) (Sigma-Aldrich). *Mtb* H37Rv (ATCC #27294) cultures were maintained in Middlebrook 7H9 broth medium supplemented with 0.2% glycerol, 0.05% Tween 80, and 10% ADC at 37°C with 5% CO_2_. Bacterial colonies were grown on Middlebrook 7H10 agar (Sigma-Aldrich) supplemented with 0.5% (vol/vol) glycerol, 0.05% Tween-80, and 10% Middlebrook oleic acid-albumin-dextrose-catalase (OADC) (Sigma-Aldrich). BDQ and Q203 were purchased from MedChem Express (Monmouth Junction, NJ, USA) and freshly dissolved in 100% dimethyl sulfoxide (DMSO; Sigma-Aldrich). Compound TBAJ-876 was synthesized by Bioduro LLC (Beijing, China) as described previously (34).

### Antimycobacterial activity and minimum inhibitory concentration determination

The growth inhibition dose-response assay was carried out using the broth microdilution method as described previously (35). Each well of clear 96-well flat-bottom Costor cell culture plates (Corning, NY, USA) was filled with 100 µL of complete 7H9 medium. BDQ/compound was added to the first well of the row to create two times the desired highest final concentration. Subsequently, a 16-point, twofold serial dilution was done, starting from the first well. *M. bovis* BCG was grown to mid-exponential phase and then diluted to an OD_600_ of 0.05 in all the wells. Plates were incubated at 37°C on an orbital shaker set at 110 rpm for 5 days. At the end of the incubation period, the culture in all wells was manually resuspended, and the OD_600_ was read using a TECAN Infinite Pro 200 plate reader. The MIC_50_ reported represents the concentration that inhibits 50% of growth compared to drug-free control.

### Bacterial killing assay

*M. smegmatis* mc (2) 155 and *M. bovis* BCG cultures were grown to exponential phase, diluted to an OD_600_ of 0.005, and aliquoted onto T-25mm^2^ tissue culture flasks (Corning, New York, USA). Test compounds were dispensed into each flask and incubated at 37°C with shaking at 110 rpm for 5–6 days. Approximately 10 µL of culture was taken out of each flask, followed by a serial dilution with PBS. Twenty-five microliters of cultures of respective dilutions was plated on each quadrant of 7H10 agar plates. *M. smegmatis* mc (2) 155 strain on the agar plates was incubated at 37°C for 3 days, and *M. bovis* BCG on the agar plates was incubated at 37°C for 10 days. Bacterial viability was determined by counting the colony-forming units (CFUs).

### Intracellular efficacy of compounds in *M. tuberculosis* H37Rv-infected THP-1 cells

THP-1 cells (American Type Culture Collection, Manassas, VA, USA) were grown to reach confluence in Roswell Park Memorial Institute (RPMI) media (Sigma-Aldrich) under standard cell culture conditions (T75-tissue culture flask, 37°C, 5% CO_2_). Confluent THP-1 cells were then treated with 200 nM phorbol myristate acetate (Sigma-Aldrich) and distributed at a density of 3 × 10^6^ cells per well in 24-well plates. After 24 h of differentiation, the cell monolayers were infected with *Mtb* at a multiplicity of infection of 10 for 60 min. Pre-warmed complete RPMI medium, with or without the test drugs, was added. BDQ at 2 µM was used as a positive control, whereas the DMSO-treated group served as a drug-free control. Mycobacterial viability was determined after 5 days of infection by CFU determination on agar plates.

### Microsomal stability assay

The microsomal stability assay was outsourced and performed by Bioduro-Sundia Ltd., USA, based on the following protocol described previously (36, 37). Working solutions of each compound are prepared from a 10 mM stock solution in DMSO diluted to a final concentration of 100 µM in 0.05 M phosphate buffer (pH 7.4). Aliquots of liver microsome working solution are transferred into 1.1 mL tubes using a multichannel pipette. Positive control (five mixed) and test compound working solutions are transferred into the tubes. The mixtures are vortexed gently and then pre-incubated at 37°C. Then, 5 mM NADPH or LM buffer (no NADPH buffer) is aliquoted into the tubes using a multichannel pipette and vortexed gently. At each time point of 0, 5, 15, 30, and 60 min with NADPH or 0, 30, and 60 min without NADPH, an aliquot is removed from each tube. Terfenadine/tolbutamide in ACN/MeOH (1:1, vol/vol) is added to quench and precipitate the microsomal incubations. Samples are capped, vigorously vortexed, and then centrifuged at 4°C. An aliquot of each supernatant is transferred for liquid chromatography using a tandem mass spectrometer (LC-MS/MSC) analysis. The MS detection is performed using a SCIEX API 4000 Q trap instrument. Each compound is analyzed by reversed phase HPLC using a Kinetex 2.6μ C18 100 Å column (3.0 mm × 30 mm, Phenomenex). Mobile phase—solvent A: water with 0.1% formic acid; solvent B: ACN with 0.1% formic acid. The amount of parent compound is determined on the basis of the peak area ratio (compound area to injected sample [IS] area) for each time point. The estimation of Cl_int_ (in µL/min/mg protein) is calculated using the following equation: CL_int_ (µL/min/mg protein) = ln(2) × 1,000 / t1 / 2 / protein conc.

### Production of *M. smegamatis* IMVs

In order to purify the IMVs of *M. smegmatis* and *M. bovis* BCG, cells were grown overnight at 37°C in 7H9 medium supplemented with 10% ADC, 0.5% glycerol, and 0.05% Tween-80 until they reached an optical density (OD) of 0.6–0.7 at 600 nm. The culture was expanded in 200 mL of supplemented 7H9 medium and grown in 1 L shake flasks (180 rpm) until it reached an OD of 0.6–0.7. This culture was used to inoculate a 500-mL culture that was then grown overnight in 2 L shake flasks (180 rpm) until it reached an OD of 0.6–0.7. Approximately 5 g (wet weight) of WT *M. smegmatis*, *M. smegmatis* I66M *c*-subunit mutant strain, and *M. bovis* (BCG) was resuspended in 20 mL membrane preparation buffer (50 mm (3-(N-morpholino)propanesulfonic acid) (MOPS), 2 mm MgCl_2_, pH 7.5) containing EDTA-free protease inhibitor cocktail (one tablet per 20 mL buffer, Roche) and 1.2 mg·mL^−1^ lysozyme. The suspension was stirred at room temperature for 45 min and additionally supplemented with 300 µL of 1 M MgCl_2_ and 50 µL DNase I, and stirring was continued for another 15 min at room temperature. All subsequent steps were performed on ice. Cells were lysed by three passages through an ice-cooled microfluidizer (model M-110L; Microfluidics, Westwood, MA, USA) at 18,000 psi. The suspension containing lysed cells was centrifuged at 4,200 × *g* at 4°C for 20 min. The supernatant containing the membrane fraction was further subjected to ultracentrifugation at 45,000 × *g* at 4°C for 1 h. The supernatant was discarded, and the precipitated membrane fraction was resuspended in membrane preparation buffer containing 15% glycerol, separated into aliquots, snap-frozen, and stored at −80°C. The concentrations of the proteins in the vesicles were determined by the bicinchoinic acid assay (Pierce, Rockford, IL, USA). IMVs were stored at −80°C.

### ATP-driven proton translocation

ATP-driven proton translocation into *M. smegmatis* IMVs was measured on the basis of a decrease in 9-amino-6-chloro-2-methoxyacridine (ACMA) fluorescence using a Cary Eclipse fluorescence spectrophotometer (Varian Inc., Palo Alto, CA, USA) as described previously (38). IMVs (0.18 mg·mL^−1^) were pre-incubated at 37°C in 10 mm HEPES/KOH (pH 7.5), 100 mm KCl, and 5 mm MgCl_2_ containing 2 µM ACMA, and a baseline was obtained by monitoring for 5 min. The reaction was started by adding 2 mM ATP or 2 mM NADH. After 8 min, any proton gradient was collapsed by the addition of 2 µM of the uncoupler SF6847 (Alexis Corporation, Lausen, Switzerland). The excitation and emission wavelengths were 410 and 480 nm, respectively.

### ATP synthesis assay

ATP synthesis was measured in flat-bottomed white 96-well microtiter plates (Corning). The reaction mix (50 µL) comprised assay buffer (50 mm MOPS, pH 7.5, 10 mM MgCl_2_) containing 10 µM ADP, 250 µM P_i_, and 1 mM NADH. The concentration of P_i_ was adjusted by adding 100 mM KH_2_PO_4_ to the assay buffer. ATP synthesis was started by adding IMVs containing *M. smegmatis* WT, *M. bovis* BCG, and the I66M *c*-subunit mutant to a final concentration of 5 µg·mL^−1^. The reaction mixture was incubated at room temperature for 30 min before adding 50 µL of CellTiter-Glo reagent, followed by incubation for another 10 min in the dark at room temperature. The luminescence produced, which correlates with the amount of ATP synthesized, was measured by an Infinite 200 Pro plate reader (Tecan), using the following parameters: luminescence; integration time, 500 ms; and attenuation, none.

### Reconstitution and ATP synthesis of mycobacterial F-ATP synthase

Recombinant *M. smegmatis* WT F-ATP synthase was purified following the protocol in Saw et al. (39). The purified enzyme was reconstituted into small unilamellar vesicles, which were generated from phosphatidylcholine type II S soybeans (Sigma-Aldrich, Steinheim, Germany), as described recently (40). Proteoliposomes containing the reconstituted F-ATP synthase were collected by centrifugation (Beckman Optima L90-K, 70.2 Ti rotor, 150,000 × *g*, 30 min), and the liposomes were resuspended in ATP synthesis buffer (100 mM Tris, 100 mM maleic acid, 5 mM MgCl_2_, 150 mM NaCl, 200 mM KCl, 5 mM KH_2_PO_4_, pH 7.5). ATP synthesis was measured at 37°C by a continuous luciferase assay, monitoring the emitted light in a luminometer (FLUOstar Omega, BMG Labtech, Ortenberg, Germany). The ATP synthesis measurement was carried out on white flat-bottomed 96-well microtiter plates. A total of 375 µL of the proteoliposomes, containing the reconstituted *Ms*F-ATP synthase and 20 µL ATP Bioluminescence Assay Kit CLS II (Roche Diagnostics, Rotkreuz, Switzerland), were mixed in the individual wells for 3 min at 37°C, and a baseline was recorded for 3 min at 37°C. After pre-incubation, ATP synthesis was started by the addition of 2 µM valinomycin (Sigma-Aldrich) to induce a ΔΨ and 5 mM ADP (final concentration each). For inhibitor studies with GaMF1.39 (90- and 180 nM) or BDQ (4 nM), proteoliposomes containing the reconstituted F-ATP synthase were additionally pre-incubated for 10 min at 4°C with the respective inhibitor before the ATP synthesis measurements were carried out.

### Generating mutant *Ms*F_1_-αβγ_H65W_

The *Ms*F_1_-αβγ_H65W_ mutant was generated via site-directed mutagenesis using the following two primers: 5′-CTG GAC TGG CCG CTG CTC GTG GAG-3′ and 5′-CAG CGG CCA GTC CAG CGC ACT GGC-3′, and pYUB1049-*Ms*F_1_-αβγ as template (41). The respective plasmid was amplified using KAPA HiFi DNA polymerase (Kapa Biosystems, Wilmington, MA, USA). PCR products were subsequently treated with DpnI prior to transformation in *Escherichia coli* TOP10 cells. Plasmids were extracted using the GeneJET Plasmid Miniprep Kit (ThermoFisher Scientific, Waltham, MA, USA), and DNA sequencing (Bio Basic Asia Pacific, Singapore) was performed to confirm the integrity of the plasmid. Plasmids carrying the gene of interest were then transformed into *M. smegmatis* mc^2^4517 electrocompetent cells for large-scale protein production and purification (42). The *Ms*F_1_-αβγ_H65W_ mutant was purified according to the protocol of WT *Ms*F_1_-αβγ described in Wong et al. (41).

### Tryptophan flurorescence spectroscopy measurements

Steady-state fluorescence measurements were performed on a Cary Varian Eclipse fluorescence spectrophotometer (Leine) using a 10-mm path length quartz cuvette. Both excitation and emission slit widths were set to 5 nm. The binding affinity of GaMF1.39 to *Ms*F_1_-αβγ_H65W_ was determined by tryptophan fluorescence quenching titration. Purified *Ms*F_1_-αβγ_H65W_ (5 µg) was titrated in 50 mM Tris-HCl, pH 7.5, and 150 mM NaCl with increasing concentrations of GaMF1.39, while quenching of tryptophan fluorescence was monitored at 320 nm following excitation at 295 nm. Dissociation constant (*K_D_*) and maximum fluorescence (ΔFmax) values were determined following the fitting of the data to an equation describing binding to a single affinity site (43).

### Molecular docking studies

Protein preparation: A homology model for *Mtb* subunit γ was generated to obtain the missing coordinates for missing γ-loop residues as described in Hotra et al. (9). Next, the assembly of α_3_, β_3_, and γ and the *c*-ring subunits was done, and the OPLS4 force field was used to verify and correct the formal charges, potentials, and bond orders of the subunits. Thus, the processed protein assembly was energy-minimized using the protein preparation module in the Schrödinger suite of programs (44). In addition, we have utilized the *Mtb* homology model generated using the prime module of the *M. smegmatis* F-ATP synthase (PDB ID: 7NJM) (19) with a γ166–179 loop in proximity to the peripheral stalk to assess the possible binding mode of GaMF1.39 at the γ-*b*′ interface.

Ligand preparation: The 3D coordinates of GaMF1.39 were prepared using ligand preparation using the OPLS4 force field. All possible conformation states at pH 7.0 were generated using the Epik Classic tool during ligand preparation, and energy was minimized using the Macromodel tool from the Schrödinger suite of programs (44).

Docking studies: The residues within 5 Å vicinity of the γ166–179 loop were used to define the site point to dock the ligands with default parameters, and the inbuilt ChemPLP and gold scoring functions in the gold program (45) were used to score and rank the molecular interactions of the ligands.

### Toxicity testing using zebrafish embryos

Zebrafish care and ethics statement: All zebrafish experiments were approved by the NTU Institutional Animal Care and Use Committee under reference no. A20038. Experiments were done using wild-type AB zebrafish. The ages of the embryos are shown in hours post fertilization (hpf). Experiment procedure: De-chorionated zebrafish wild-type embryos at 48 hpf (*n* = 120) were used. The embryos were immersed in 5 mL of E3 medium containing GaMF1.39 in a 6-well plate for 5 days. DMSO was used as vehicle control. The embryos were observed under a microscope. Abnormal phenotypes and survival rates in each treatment group were observed for 5 consecutive days. The experiment was repeated twice, with three replicates each.

### Biofilm testing

A biofilm assay was performed in a 24-well plate to evaluate the activity of BDQ and GaMF1.39 on *P. aeruginosa* PAO1 WT and *E. coli* UTI 189 biofilms. Overnight cultures of *P. aeruginosa* PAO1 WT and *E. coli* UTI 189 were diluted in M9 glucose media (1× M9 salts, 2 mM MgSO_4_, 0.1 mM CaCl_2_, 0.4% wt/vol glucose) to a final OD_600_ of 0.05. Then, 1 mL of the diluted culture was then added to each well of the 24-well plate and incubated at 37°C with 100 rpm shaking. Following 3 h of incubation, each well was washed once, and the culture media was replaced with 1 mL of 1× PBS (pH 7.4) or fresh M9 glucose medium containing 1–3 µM of BDQ or 3–9 µM of GaMF1.39. The treated samples were further incubated under the same conditions for 3 h. At *t* = 6 h, the samples were collected. The 1× PBS (pH 7.4) buffer containing suspended bacteria cells with treatment was collected into 1.5 mL Eppendorf tubes and considered to be “planktonic samples.” Subsequently, each well was washed once with 1 mL of 1× PBS (pH 7.4) before resuspending biofilm cells in the same volume of 1× PBS (pH 7.4). Biofilm cells were dislodged into the buffer by means of a cell scraper, and 1 mL of the sample was collected into 1.5 mL Eppendorf tubes labeled “biofilm samples.” The samples contained in Eppendorf tubes were sonicated in a water bath using the following settings: 5 min degas mode, 37 Hz, 100%, followed by 5 min pulse mode, 37 Hz, 100%. Subsequently, the samples are serially diluted and used for CFU counts. The experiment was repeated independently at least two times, with two technical replicates per independent experiment. CFU counts were analyzed using GraphPad Prism V9.3.0 (43) using a two-way analysis of variance and multiple comparisons of column effect (concentration of compound against untreated control) within each row (planktonic vs biofilm samples).

### Checkerboard titration assay

A checkerboard titration assay was carried out as described previously (46, 47). Briefly, GaMF1.39, which was synthesized according to Hotra et al. (9), Q203, and CFZ (Sigma-Aldrich), respectively, were added to complete 7H9 medium-containing 96-well flat-bottom Costar cell culture plates. Twofold serial dilutions were done to allow 10 different concentrations of GaMF1.39 (0.1 μM to 60 μM) to be tested for interaction with 14 different concentrations of Q203 (0.4 pM to 8 nM). Hence, a total of 140 different concentration combinations were tested between GaMF1.39 and Q203. In combination studies with CFZ, 10 different concentrations of GaMF1.39 (0.1 μM to 60 μM) were tested with 7 different concentrations of CFZ (0.03 to 2 µM). Each 96-well plate had a 7H9 medium-only control well and a drug-free bacterial culture control well. *M. bovis* BCG was cultured in complete 7H9 medium and grown to mid-exponential phase. Subsequently, the culture was diluted to an OD_600_ of 0.01 using complete 7H9 medium and added to each well in the 96-well plate to create a final OD_600_ value of 0.005. The plates were incubated for 6 days at 37°C. After the incubation period, the culture in each 96-well plate was manually resuspended, and the OD_600_ of each well was read using a Tecan Infinite Pro 200 plate reader. A calculation of the fractional inhibitory concentration index (FICI) was done to analyze the results. The FICI is calculated as (MIC of drug A in combination / MIC of drug A alone) + (MIC of DARQ B in combination / MIC of DARQ B alone). This calculation was done only for wells that showed 50% inhibition of bacterial culture growth compared to drug-free bacterial culture wells. A FICI of ≤0.5 indicates synergy, a FICI of >0.5 to 4 indicates additivity (no interaction), and a FICI of >4 indicates antagonism (48).

## SUPPLEMENTAL MATERIAL

Supplemental material spectrum.02282-23-s0001.pdf Fig. S1 to S4 and Table S1.

ASM does not own the copyrights to Supplemental Material that may be linked to, or accessed through, an article. The authors have granted ASM a non-exclusive, world-wide license to publish the Supplemental Material files. Please contact the corresponding author directly for reuse.
