# Supplementary material for: GaMF1.39’s antibiotic efficacy and its enhanced antitubercular activity in combination with clofazimine, Telacebec, ND-011992, or TBAJ-876
Source: Microbiol Spectr. 2023 Nov 20;11(6):e02282-23. doi: 10.1128/spectrum.02282-23 (PMC10715162; doi:10.1128/spectrum.02282-23)
Supplement: Supplemental material — Fig. S1 to S4 and Table S1. [file spectrum.02282-23-s0001.pdf]

GaMF1.39's antibiotic efficacy and its enhanced antitubercular activity in combination with Clofazimine, Telacebec, ND-011992 or TBAJ876

Priya Ragunathan[1], Pearly Shuyi Ng[2], Samsher Singh[3], Wee Han Poh[4], Dennis Litty[5], Nitin Pal Kalia[6], Simon Larsson[3], Amaravadhi Harikishore [1,7], Scott A. Rice[1,4#], Philip W. Ingham[3], Volker Müller[5], Garrett Moraski[8], Marvin J. Miller[8], Thomas Dick[9-11], Kevin Pethe[1,3,12], and Gerhard Grüber[1,*]

[1]School of Biological Sciences, Nanyang Technological University, 60 Nanyang Drive, Singapore 637551, Republic of Singapore

[2]Experimental Drug Development Centre, Agency for Science Technology and Research, A*STAR, 10 Biopolis Road, Singapore 138670, Republic of Singapore

[3]Lee Kong Chian School of Medicine, Nanyang Technological University, Experimental Medicine Building, Republic of Singapore

[4]Singapore Centre for Environmental Life Sciences Engineering, Nanyang Technological University, Singapore

[5]Molecular Microbiology and Bioenergetics, Institute of Molecular Biosciences, Johann Wolfgang Goethe University Frankfurt/Main, Max-von-Laue-Str. 9, 60438 Frankfurt, Germany

[6]Department of Biological Sciences (Pharmacology & Toxicology), National Institute of Pharmaceutical Education and Research, Hyderabad, Telangana, India 500037

[7]School of Chemistry, Chemical Engineering and Biotechnology, Nanyang Technological University, 21 Nanyang Link, Singapore 637371, Republic of Singapore

[8]Department of Chemistry and Biochemistry, University of Notre Dame, Notre Dame, Indiana, USA

[9]Center for Discovery and Innovation, Hackensack Meridian Health, 111 Ideation Way, Nutley, NJ 07110, USA

[10]Department of Medical Sciences, Hackensack Meridian School of Medicine, 123 Metro Boulevard, Nutley, NJ 07110, USA

[11]Department of Microbiology and Immunology, Georgetown University, 3900 Reservoir Road NW Medical-Dental Building, Washington, DC 20007, USA

[12]National Centre for Infectious Diseases (NCID), 16 Jalan Tan Tock Seng, Singapore 308442

# Current address: Microbiomes for One Systems Health and Agriculture and Food, CSIRO, Westmead NSW, Australia

* To whom correspondence may be addressed: Prof. Dr. Gerhard Grüber, Tel.: + 65 – 6316 2989, E-mail: ggrueber@ntu.edu.sg

Running title: GaMF1.39, a potent anti-TB inhibitor

Keywords: Bioenergetics, *Mycobacterium tuberculosis*, tuberculosis, F-ATP synthase, oxidative phosphorylation, anti-TB compound

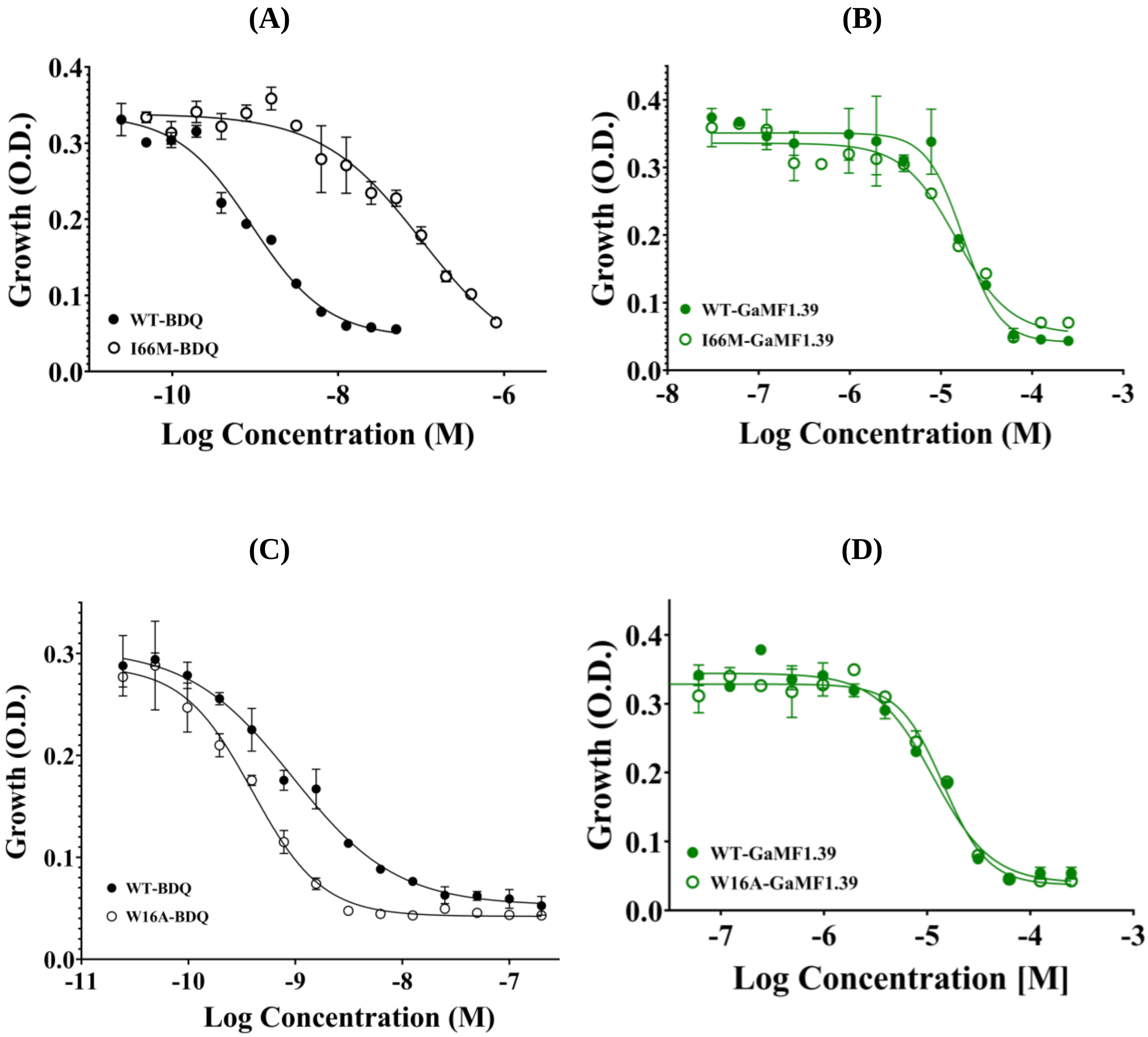

Supplementary Figure S1: (*A*) Growth inhibition by BDQ on WT *M. smegmatis* and the I66M *c*-subunit mutant strain or (*B*) by GaMF1.39. Comparison of growth inhibition on WT *M. smegmatis* cells by BDQ (*C*) or (*D*) GaMF1.39 and BDQ. Three independent experiments were carried out, each with three technical replicates. Error bar indicates standard deviation.

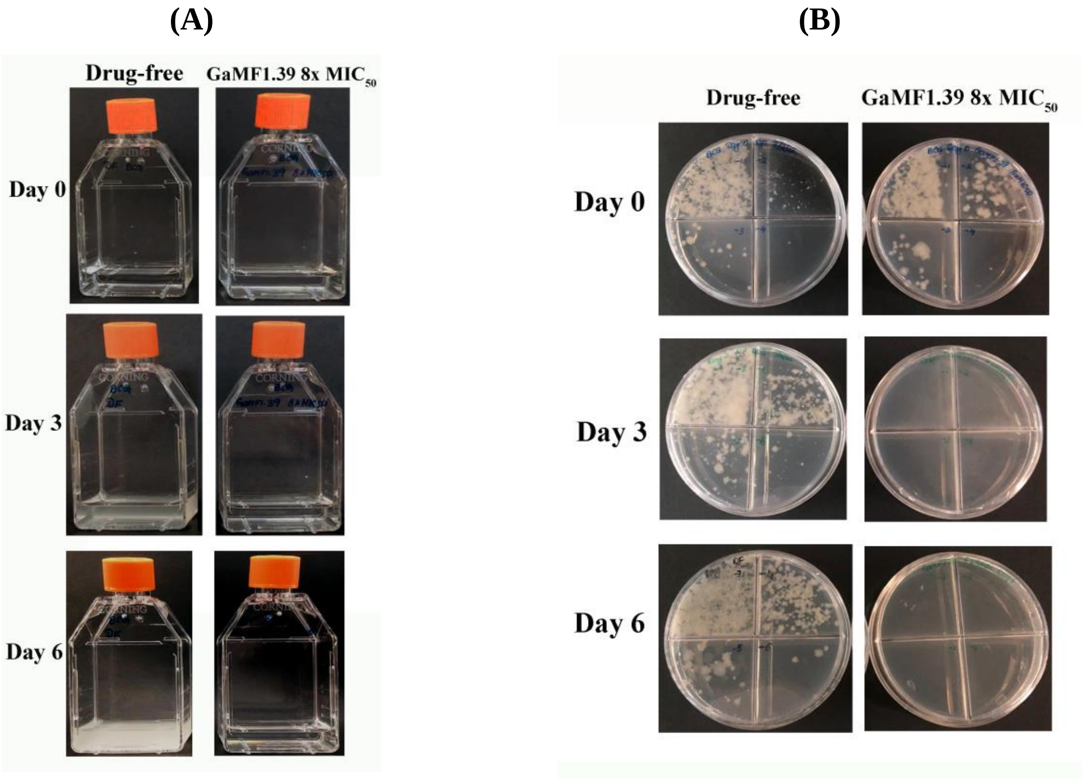

Supplementary Figure S2: Initial six days of GaMF1.39 kill kinetics against *M. bovis* (BCG). The bacteria were grown in liquid culture (LBT) (A) in the presence of the indicated concentrations of GaMF1.39 up to 6 days. CFU was calculated by plating the culture on 7H10 agar plates (B).

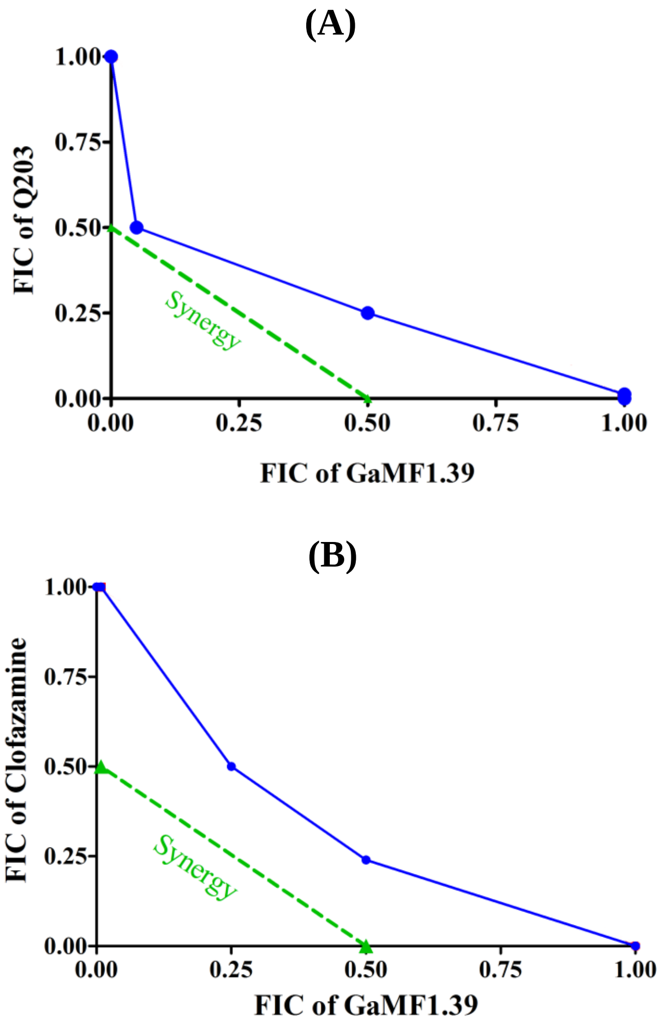

Supplementary Figure S3: Isobolograms of the combination of GaMF1.39 and Q203 (*A*) or GaMF1.39 and Clofazamine (*B*) against *M. bovis* BCG. The green dash line indicates ideal isobole, where drugs act in synergy and independently. An FICI of ≤0.5 indicates synergy, a FICI of >0.5 to 4 indicates additivity (no interaction), and an FICI of >4 indicates antagonism[1].

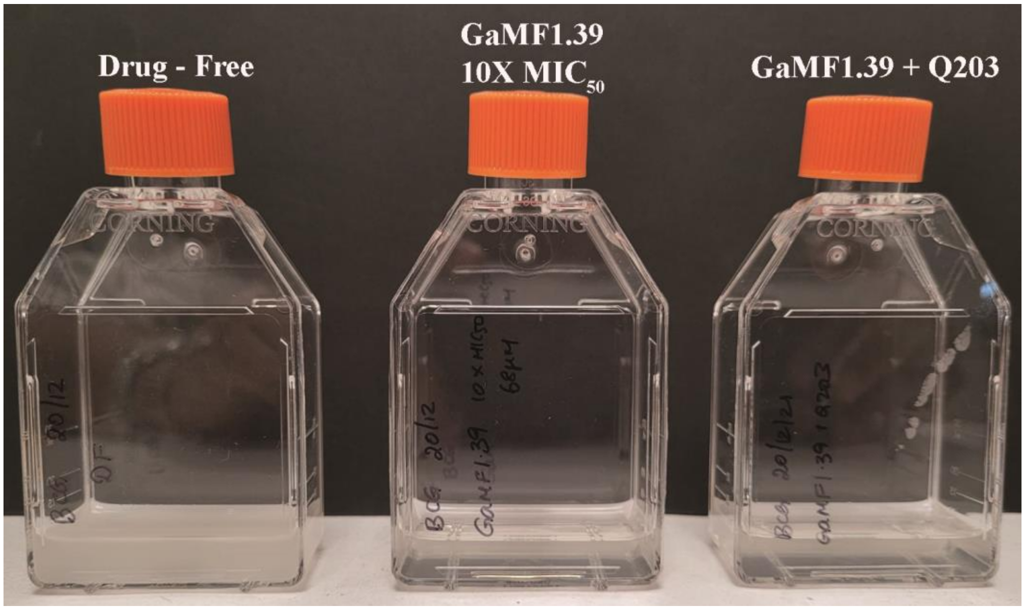

Supplementary Figure S4: *M. bovis* cultures grown for killing kinetics experiment in the absence of any drug, in the presence of GaMF1.39 (10x MIC_{50}) and GaMF1.39 (10x MIC_{50}) + Q203 (100 nM). The picture of the flasks were taken at t=10 days showing the killing potency of GaMF1.39 and GaMF1.39 + Q203.

Drug A	Combination with drug B	MIC				FICI[b]	Outcome
		Drug A alone	Drug A in combination	Drug B alone	Drug B in combination		
GaMF1.39	Q203	6.8 µM	0.23 µM	1 nM	0.5 nM	0.55	Additivity
GaMF1.39	Clofazamine	6.8 µM	3 µM	0.25 µM	0.125 µM	0.75	Additivity

Supplementary Table 1: The FICI was calculated as (MIC of drug A in combination/MIC of drug A alone) + (MIC of drug B in combination/MIC of drug B alone)[2]. An FICI of ≤0.5 indicates synergy, a FICI of >0.5 to 4 indicates additivity (no interaction), and an FICI of >4 indicates antagonism[1].

References

1. Odds, F. C. (2003) Synergy, antagonism, and what the chequer board puts between them. *J. Antimicrob. Chemother.* 52:1. doi:10.1093/jac/dkg301.
2. Hsieh, M. H., Yu, C. M., Yu, V. L., and Chow, J. W. (1993) Synergy assessed by checkerboard. A critical analysis. *Diagn. Microbiol. Infect. Dis.* 16, 343–349.
